# The New Pharmaceutical Compositions of Zinc Oxide Nanoparticles and Triterpenoids for the Burn Treatment

**DOI:** 10.3390/ph13090207

**Published:** 2020-08-22

**Authors:** Nina Melnikova, Olga Vorobyova, Alyona Balakireva, Darina Malygina, Anna Solovyeva, Kseniya Belyaeva, Dmitry Orekhov, Alexander Knyazev

**Affiliations:** 1 Department of Pharmaceutical Chemistry, Privolzhsky Research Medical University, 10/1 Minin sq., 603950 Nizhny Novgorod, Russia; vorobeva_olga1990@mail.ru (O.V.); Liza200000@yandex.ru (A.B.); mds73@yandex.ru (D.M.); sannag5@mail.ru (A.S.); kseniyabelyaeva2018@bk.ru (K.B.); 2 Department of Engineering Physics and Chemistry, Nizhny Novgorod State Technical University n.a. R.E. Alekseev, 24 Minin st., 603950 Nizhny Novgorod, Russia; mitriy07@mail.ru; 3 Faculty of Chemistry, Lobachevsky University, 23/5 Gagarin Av., 603950 Nizhny Novgorod, Russia; knyazevav@gmail.com

**Keywords:** ZnO NPs, lupane triterpenoids, oleogels, treatment of burns

## Abstract

We studied oleogels containing zinc oxide nanoparticles (ZnO NPs) and lupane triterpenoids in sunflower oil for the treatment of burns. The modification of ZnO was carried out by treatment with alcohol solutions of betulin, betulonic acid, betulin diacetate and betulin diphosphate. The properties of modified ZnO NPs were studied by powder XRD (average sizes of 10–20 nm), FTIR (νZnO 450 cm^−1^), UV–vis (345–360 nm), and blue–violet emission (380–420 nm). The identification and assay of modified ZnO NPs and triterpenoids were estimated. The treatment by oleogels of deep II-degree burns was studied on rats using histological studies, Doppler flowmetry and evaluation of enzymes activity and malonic dialdehyde (MDA) level. After the action of oleogels, burn wound area, and the necrosis decreased twice on the 10th day in comparison with the 1st day after burn. The microcirculation index in the near-wound zone by 20–30% improved compared with the group without treatment. Evaluation of the enzyme activity and the MDA level after treatment by oleogels during the course of 10 days showed them returning to normal. The improvement of antioxidant biochemical indexes, as well as wounds’ healing, was mainly determined by the influence of zinc oxide nanoparticles.

## 1. Introduction

The topical treatment of a thermal burn wound mainly consists of reducing inflammation and bacterial colonization in the wound, reducing scarring and reparative tissue regeneration [1,2,3]. The wound healing depends on collagen formation [4], metabolism and immune status of the organism in general.

Recently, zinc oxide nanoparticles, capable of exhibiting bactericidal and bacteriostatic effects, as well as promoting collagen synthesis, are of great interest as components of anti-burn agents [5,6,7]. The antibacterial effect of zinc oxide nanoparticles, as well as other metal nanoparticles, is explained by the ability of zinc oxide nanoparticles to effectively penetrate the bacterial cell membrane, causing the generation of reactive oxygen species, leading to apoptosis [8]. The effect of zinc oxide nanoparticles on wound healing is attributed to the effects of zinc as an essential trace element that controls many biological processes. Zinc is a component of more than 300 metalloenzymes and over 2000 transcription factors that are needed for regulation of lipid, protein and nucleic acid metabolism, and gene transcription via factors possessing the zinc-finger motifs [5]. Zinc also plays an essential role in maintaining the proper reproductive function, immune status, and wound repair via regulation of DNA and RNA polymerases, thymidine kinase, and ribonuclease [6]. Early on, the role of zinc in collagen metabolism in the skin of zinc-deficient rats was studied in work [9]. The importance of zinc as a component of drugs for therapy in dermatology, among them wound healing, was shown in review [6]. In particular, the efficiency of zinc was shown using (50 mg) in burn treatments in many burn centers across North America [10].

The advantages of zinc oxide nanoparticles having constant valency of zinc ions are high aggregate stability, lower toxicity with the same high permeability through the skin and lipid membranes in comparison with gold and silver nanoparticles [7,11]. The large specific surface of the nanoparticles allows other anti-burn drugs to adsorb and include into nanoparticles.

Plant triterpenoids, such as derivatives of betulinic acid, asiatic acid, madecassic acid and others, also contribute to the activation of collagen synthesis necessary for effective wound healing [12,13,14,15,16]. The collagen-proliferative properties of these compounds are due to an increase in collagen concentration in the wound [17]. Modification of the zinc oxide nanoparticle surface by triterpenoids, which activate collagen synthesis, can cause a synergistic effect of regeneration of burned skin.

One of the wound healing drugs for the regeneration of I-II-degree burns is Episalvan in the dosage form of oleogel containing birch bark dry extract in sunflower oil [15]. The dry extract of the dosage form includes triterpenoids, such as betulin (up to 80%), betulinic acid (up to 4%), oleanolic acid, which affect collagen synthesis and exhibit a complex of anti-inflammatory, anti-tumor, hepatoprotective and other properties.

It can be proposed that a combination of triterpenoids (tissue repair agents) with zinc oxide nanoparticles with high permeability through skin tissues provides synergism of topical action. Zinc ions influence metabolism, homeostasis, the immune system and antioxidant enzyme defense expands wound-healing effect. Therefore, the combined effect of oleogel components at the systemic level in the human body topically may provide high treatment efficiency in deep burn wounds.

The work considers the developed new anti-burn drugs with zinc oxide nanoparticles and triterpenoids from the group of betulin and its derivatives. For this purpose, we studied: (a) the synthesis of zinc oxide nanoparticles with an average size of not more than 50 nm and their modification with triterpenoids; (b) optimization of the oleogel dosage form composition; (c) the study of the effectiveness of the developed oleogel in an experiment on a model of a deep II-degree burn wound on rats based on morpho-histological analysis, microcirculation index, antioxidant enzyme activity (superoxide dismutase, catalase, glutathione reductase, glucose-6-phosphate dehydrogenase, lactate dehydrogenase) and malonic dialdehyde level.

**Figure 1:**
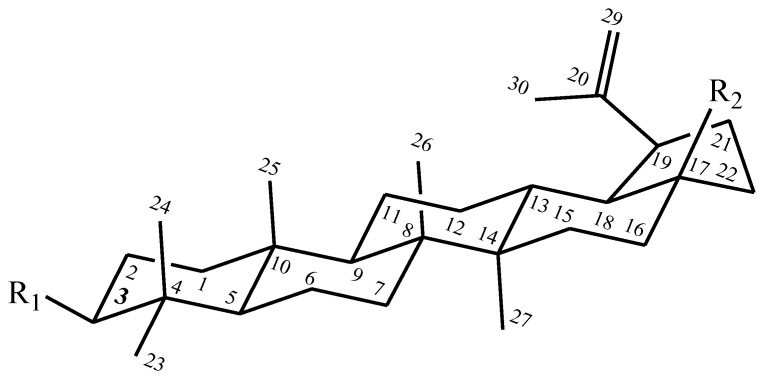
Formulas of betulin and its derivatives. betulin: R_1_—OH; R_2_—CH_2_OH; betulonic acid: R_1_—=O; R_2_—COOH; betulin 3,28-diphosphate: R_1_—OPO_3_H_2_; R_2_—CH_2_OPO_3_H_2_; betulin 3,28-diacetate: R_1_—OCOCH_3_; R_2_—CH_2_OCOCH_3_.

In this study, betulin (B), betulonic acid (BA), betulin diacetate (BDA) and betulin diphosphate (BDP) were used. The general formulas are shown in Figure 1.

## 2. Results and Discussion

### 2.1. Properties of Zinc Oxide Nanoparticles Modified by Triterpenoid

Zinc oxide nanoparticles (ZnO NPs) were prepared by the sol-gel method using zinc acetate as a precursor and lithium or sodium hydroxide as a precipitant in ethanol. The sol–gel process was carried out with the replacement of a solvent (ethanol–heptane); after that, nanoparticles were separated and dried [18,19]. The modification of nanoparticles (ZnO NPs) was carried out by treatment with ethanol solutions of betulin (B), betulonic acid (BA), betulin diacetate (BDA) and betulin diphosphate (BDP) for 1 h.

**Figure 2:**
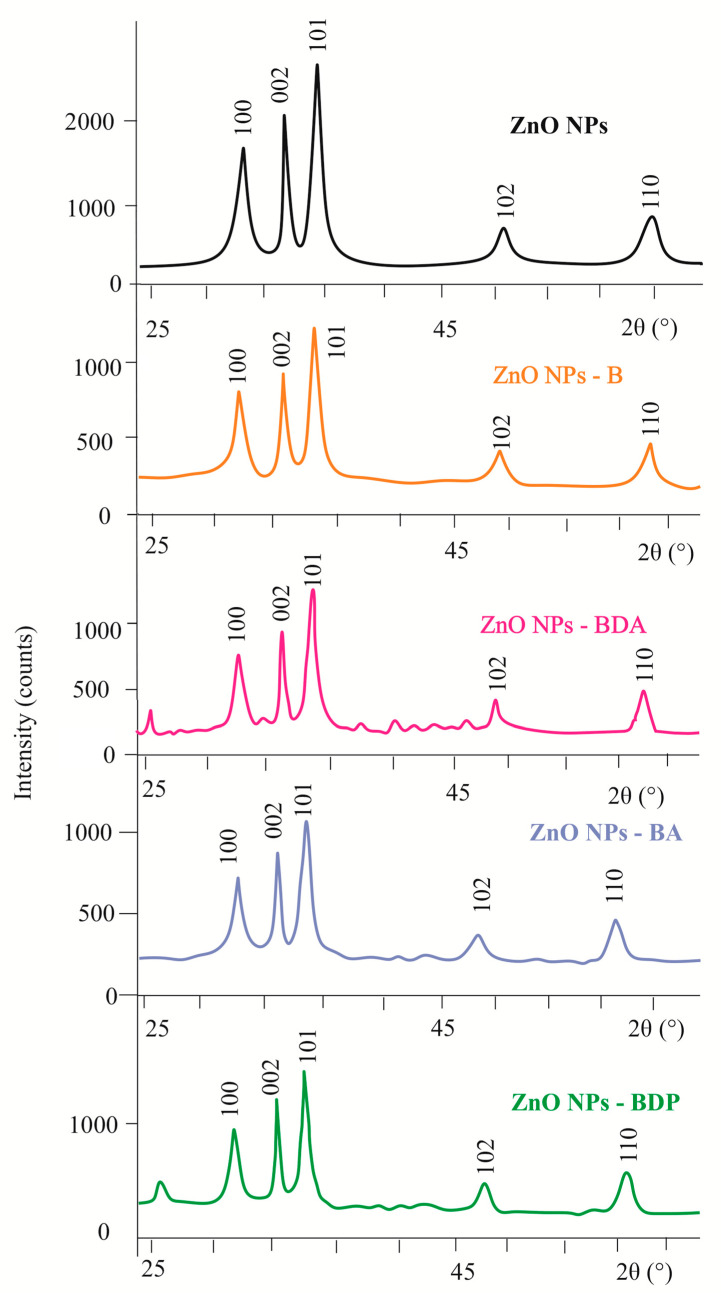
Powder XRD patterns of ZnO NPs modified by triterpenoids.

The structure of dry ZnO NPs modified by triterpenoids, as well as the initial ZnO NPs, corresponded to the hexagonal structure of wurtzite according to powder X-Ray diffraction (XRD) patterns (Table 1, Figure 2). The average powder size (D) was estimated using the Scherrer Equation (1) from the powder XRD pattern.

(1) $$D=\frac{k\lambda }{\beta \mathrm{Cos}\theta },$$

where λ is the wavelength of the X-ray and equals 1.5056 Å, k is 0.89, β is the half-peak width of the diffraction peak, and θ is the Bragg angle. The Bragg scattering angles 2θ (100, 002, 101, 102, and 110) of the modified nanoparticles coincided with the values of the initial zinc oxide nanoparticles.

According to Equation (1), the average diameter of the five samples was calculated to be from 11.5 to 22.6 nm (Table 1). Thus, these ZnO particles were, in fact, nanoparticles.

**Figure 3:**
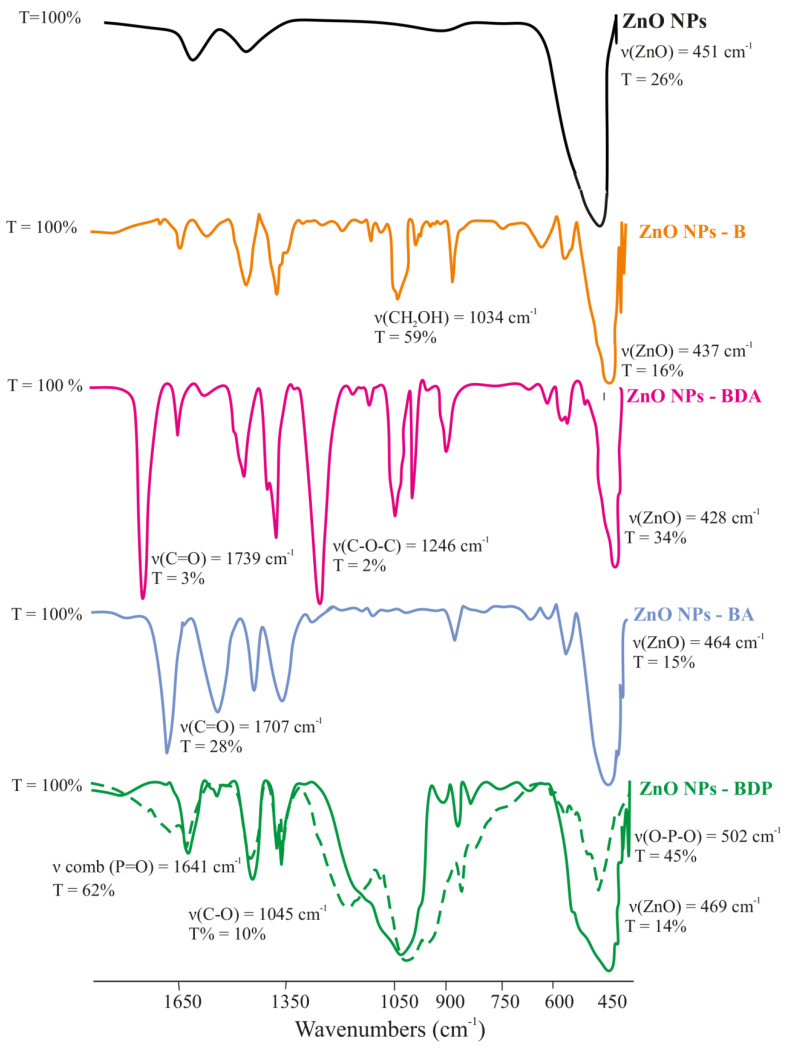
FTIR spectra of zinc oxide nanoparticles and complexes of zinc oxide nanoparticles with adsorbed triterpenoids (dashed line is FTIR spectrum of BDP).

The intense absorption band, which is characteristic of Zn–O stretching vibrations (ν 500–450 cm^−1^), was observed in the FTIR spectra of all modified samples of ZnO NPs. The immobilization of triterpenoids on the surface of zinc oxide nanoparticles was confirmed by the presence in the spectra of stretching vibration bands characteristic of the studied triterpenoids (Table 2, Figure 3).

The most significant changes in the FTIR spectra of the modified nanoparticles were observed in samples of ZnO NPs-BDP. The immobilization of BDP into the surface layer of ZnO NPs confirms the presence in the spectrum of absorption bands characteristic of stretching vibrations of hydroxyl groups (3447 cm^−1^), CH, CH_2_, CH_3_ (2948 and 2875 cm^−1^); phosphoryl group P=O (1641 cm^−1^); P–O and C–O groups (1193, 1033, 983, 971 cm^−1^), phosphate groups in the region of 500–600 cm^−1^.

The difference in ZnO NPs-BDP spectrum is the change in the complex band in the region of 450–500 cm^−1^, the intensity of which is two times higher than in the spectrum of the BDP. Significant differences also occurred in the region of 1250–980 cm^−1^, in which an intense broad band was observed, including several undivided bands (Figure 3).

**Figure 4:**
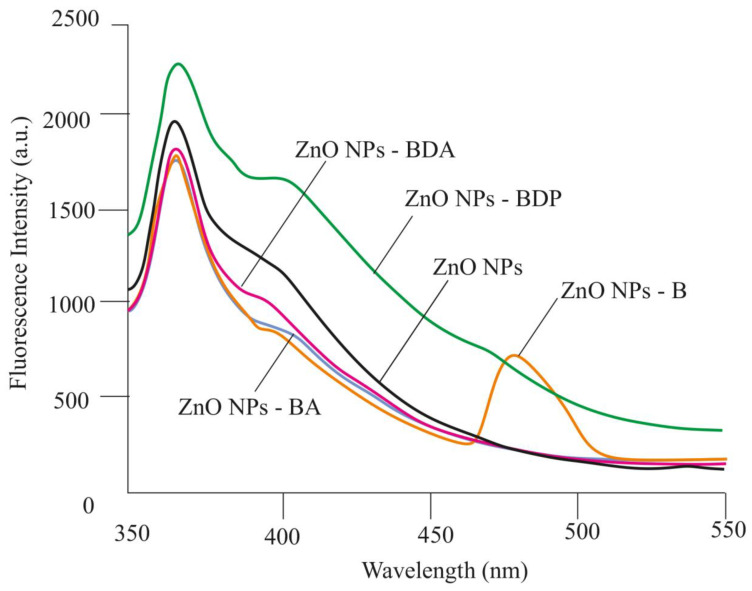
Fluorescence emission spectra of ZnO NPs and ZnO NPs modified by triterpenoids.

Fluorescence spectra of dispersions of nanoparticles (ZnO NPs-B, ZnO NPs-BDA, ZnO NPs-BA, ZnO NPs-BDP) were recorded in ethanol (Figure 4). All modified nanoparticles in ethanol, such as the initial zinc oxide nanoparticles, showed intense blue–violet emission in the range 365–420 nm due to exciton radiation in the near field (Figure 4).

A similar blue–violet emission of ZnO NPs (λ_em_ = 411 nm at λ_ex_ = 320 nm) was studied by Efafi B. [20]. The authors attribute this effect to the density of oxygen vacancies decreasing due to acetone used as a solvent in the synthesis. Likely, the immobilization of betulin derivatives on the surface of ZnO NPs during the modification process is also associated with a decrease in the density of crystalline defects caused by oxygen.

The same violet–blue emission in the region of 360–400 nm was previously observed in fluorescence spectra of ZnO NPs obtained by the sol–gel method in the presence of triethanolamine in zinc acetate sol [21].

The influence of the ZnO NPs synthesis and their modification by triterpenoids on blue emission in fluorescence spectra can be explained using data of the authors [22]. The authors showed the correspondence of theoretically calculated Gaussian fluorescence spectra and diagrams of the energy zones of ZnO NPs obtained by various methods in different media. Theoretical spectra were calculated, taking into account the width parameters (the shoulder and unresolved bands) of each emission band: 432, 382, 404, 412, 408 and 374 nm. It is believed that violet emission usually occurs due to interstitial defects of zinc (Zn_i_) during the electronic transition from the level of the Zn_i_ site to the valence band, and blue emission is associated with the transition from the Zn_i_ level to vacant defects [21,23,24,25].

We believe that the manifestation of violet emission in the region of 365–410 nm by the zinc oxide nanoparticles is due to the same reasons such as a higher concentration of grains, dislocations, and surface traps, and additionally adsorbed triterpenoids.

**Figure 5:**
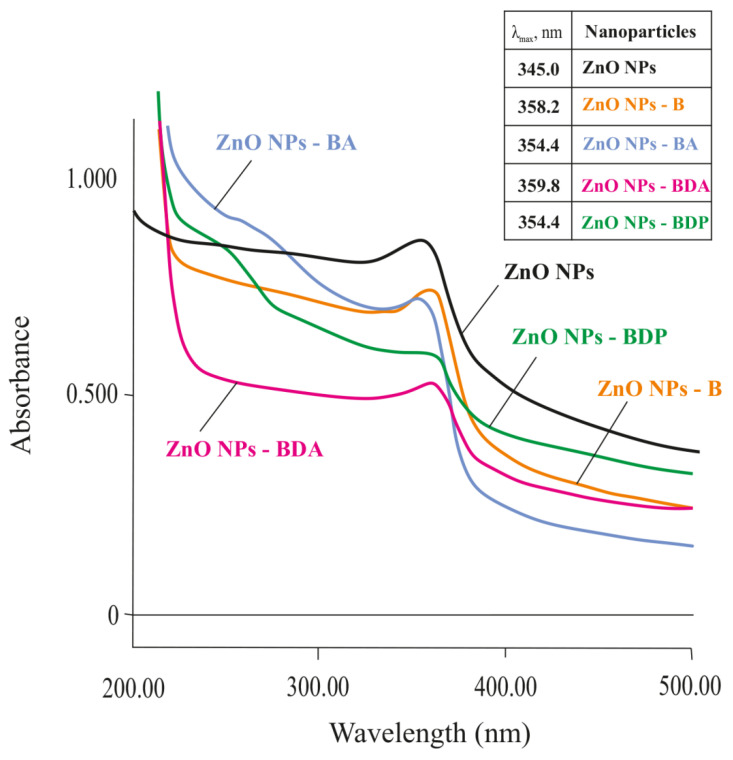
UV–Vis spectra of ZnO NPs and ZnO NPs modified by triterpenoids. The data of λ_max_ are presented in the insert.

Blocking of zinc interstitial defects due to the adsorption of betulin derivatives should reduce the laser ablation effect, which manifests itself as an absorption band in the UV spectrum in the region of 350–370 nm (Figure 5). The effect of triterpenoids on the “quenching” of this effect was evaluated by UV spectral analysis on the example of modification of ZnO NPs by triterpenoids such as betulin (B), betulonic acid (BA), betulin diacetate (BDA) and betulin diphosphate (BDP).

The study of the physicochemical properties of modified zinc oxide nanoparticles allows us to propose the identification of nanoparticles as an active pharmaceutical ingredient. There are FTIR spectrum in the range 4000–400 cm^−1^, band in the region 340–360 nm with λ_max_ in UV absorption spectrum in ethanol or methanol, emission in the region 365–450 nm in the photoluminescence spectrum, and the average diameter of wurtzite particles is 10–20 nm (by use powder XRD pattern). The identification of immobilized triterpenoids on the surface of nanoparticles was estimated by FTIR by comparison with the spectra of the initial triterpenoids (experimental). Assay of triterpenoids was carried out by HPLC after desorption in ethanol or methanol. Figure S1 shows HPLC data of triterpenoids solution before and after inclusion on the ZnO NPs’ surface. The concentration of immobilized triterpenoids in ZnO NPs was calculated using formula (2):

(2) $$\Gamma =\frac{\left(C_{0}-C_{\tau }\right)\cdot V}{m_{\mathrm{ZnO}}}$$

where C_0_, C_τ_—triterpenoid concentration before and after sorption (mg∙ml^−1^); V—volume of triterpenoid solution (mL); m_ZnO_—a mass of ZnO NPs (g).

**Figure 6:**
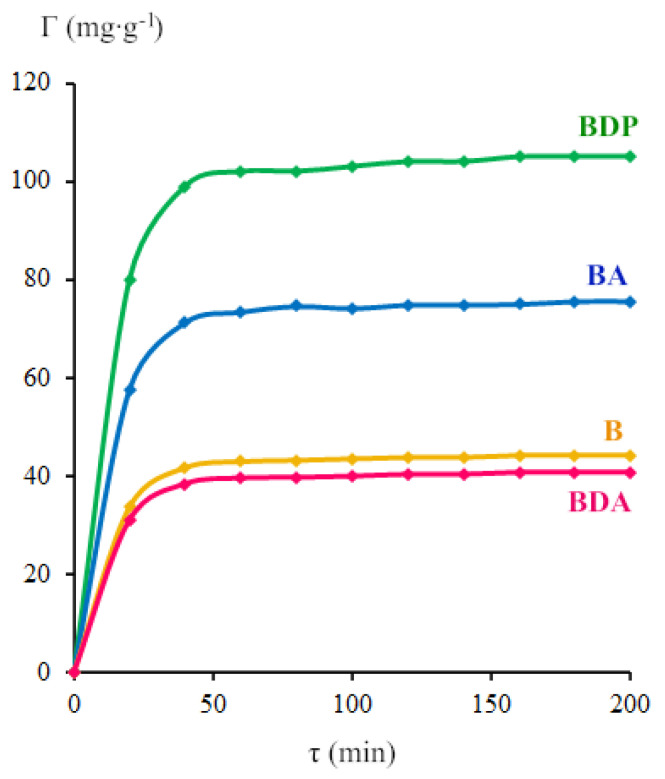
The dependence of triterpenoid surface concentration on sorption time by ZnO NPs.

Figure 6 shows the change of triterpenoid surface concentration (Γ) in time. Triterpenoid surface concentration was taken as the value on a plateau of the curve of dependence Γ = f(τ) after sorption during 1 h. The sorption of triterpenoids by zinc oxide nanoparticles changes in the order: BDA ≈ B < BA < BDP.

The obtained zinc oxide nanoparticles, both initial and modified, were used as the active pharmaceutical ingredient for the preparation of four types of oleogel samples (Table 3). Oleogels were obtained by suspending of betulin (10 g) in 70 g of sunflower oil under the action of ultrasound (44 kHz) for 10 min, after which initial or modified zinc oxide nanoparticles (5 g) were carefully added during intensive sonication. After the formation of a homogeneous mixture, the mass of oleogels was brought by the sunflower oil to 100 g and re-treated with ultrasound for 5 min.

### 2.2. Biomedical Studies

The mechanism of wound healing in rats and humans is different, since wound contraction and collagen formation are dominant in the treatment of rats, in contrast to re-epithelialization observed in humans [26]. The healing of burn wounds was studied in rats, taking into account the simplicity and economy of the model, as well as the speed of wound healing and the effect of zinc and betulin derivatives on collagen formation in various processes [9,10,27,28,29].

The name of each animal group was the same as oleogel’s name for treatment. Biomedical studies were carried out on six groups of animals (five rats in the group): burnt untreated, ZnO NPs, ZnO NPs-BDA, ZnO NPs-BA, ZnO NPs-BDP and an intact group. The treatment was carried out for 10 days, treating the wound twice a day, at a dose of 25 mg∙cm^−2^.

#### 2.2.1. Morpho-histological Studies

**Figure 7:**
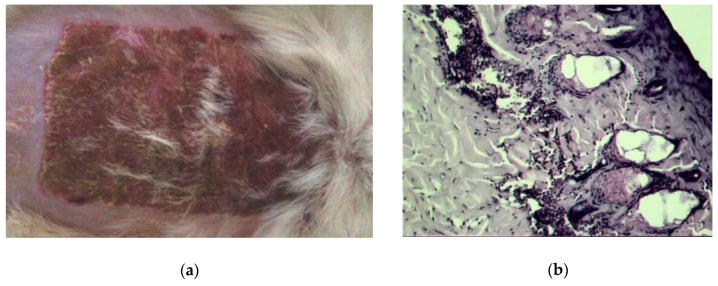
Visualization of a burn wound of burned untreated animals on the 1st day. (**a**) Image of burn wound; (**b**) Morpho-histological image of burn wound tissue (hematoxylin-eosin, ×600).

During the 1st day of the experiment, animals of all burn groups had a deep II-degree burn of the skin, and an increase in the area of burn wounds was observed in all groups by 15–20% while the initial burn area was equal to 14.0 ± 0.5 cm^2^. The skin of burn wounds was dense, insensitive to pain stimuli, and had a loose and rough scab. On the 1st day after a burn injury, the following clinical signs were noted in all animals of the control group without treatment: lethargy, refusal of feed, polydipsia. In the area of burn injury, necrotic skin tissue was tightly attached to the underlying tissue, somewhat rising above the healthy ones. On the 1st day after modeling the burn, swelling and hyperemia were observed along the edges of the wound, pronounced coagulation necrosis was shown on the damaged skin area with the formation of a scab uneven in thickness (Figure 7).

**Figure 8:**
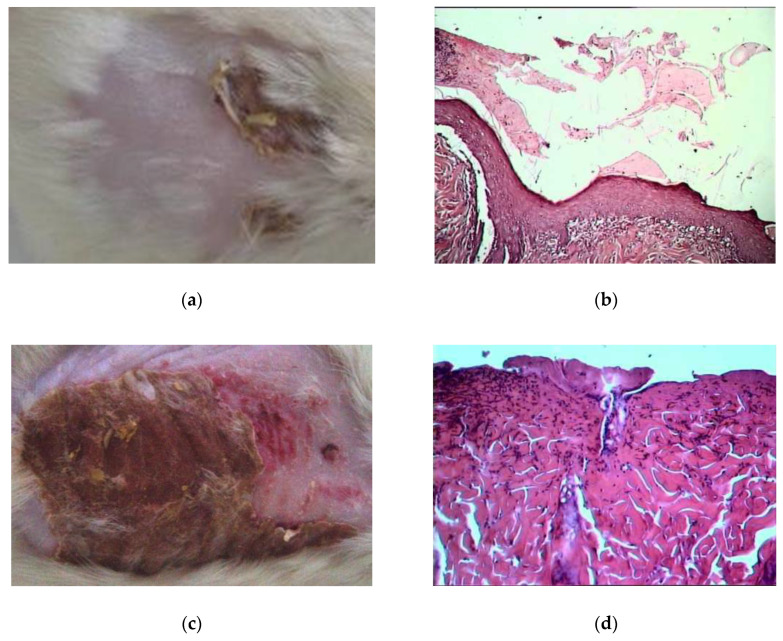
Visualization of burn wound healing on the 10th day. (**a**) Image of burn wound under ZnO NPs-BA oleogel treatment; (**b**) Morpho-histological image of burn wound tissue under ZnO NPs-BA oleogel treatment (hematoxylin-eosin, ×400); (**c**) Image of burn wound without treatment; (**d**) Morpho-histological image of the burn wound tissue without treatment (hematoxylin-eosin, ×400).

On the 10th day of treatment by oleogels, the burn wound area was decreased twice compared to the initial burn area (by 45–55%). The bottom of the burn wound was cleared of scab residues and filled with granulations. Signs of the transition of granulation tissue into connective tissue appeared and the vascular invasion was noted (Figure 8a,b). In contrast, at the same time, the burn wound area increased by 10–15% in the untreated burned group, and scab remained (Figure 8c,d).

In general, the animals treated with oleogels that had a satisfactory wool condition did not have deviations in behavior and did not lose their appetite in contrast to the control burnt group without treatment. Healing of the burn wound in the control burnt group without treatment on the 1st and 10th day was slow. The burn wound area on the 10th day decreased twice in comparison with the 1st day (Table 4).

To distinguish features of healing processes of thermal burns on the basis of therapy with ZnO NPs-triterpenoid composition, the activation of metabolic processes in antioxidant defense should be noted.

#### 2.2.2. Vasodilatation Effect of Pharmaceutical Compositions of ZnO NPs Modified by Triterpenoids

The treatment by ZnO NPs oleogels aimed to improve the microcirculation in zones of vascular disorders of tissues after thermal injury. The dynamics of microcirculation were explored using laser Doppler flowmetry. Analysis of reflected from tissue radiation is based on isolation of the recorded signal using the Doppler frequency shift of the reflected signal. The Doppler frequency shift is proportional to erythrocytes motion speed at blood flow changing in the microcirculatory system. The microcirculation index (MI) shows the middle level of perfusion (medium flow of erythrocytes) in the volume unit of tissue per unit time (Table 5).

Values of the MI also show the depth of the wound. The deficient value of the MI (less than 1 perfusion unit) corresponds to a deep wound. In the study, burned animal’s microcirculation index decreased to 6 perfusion units, as usual for deep II-degree burn.

Data of local microcirculation showed that the use of ZnO NPs–triterpenoid oleogels for 10 days improved the microcirculation index (MI) by 20–35% in the near-wound zone in comparison to MI of control burned group.

In general, on the 10th day of the post-burn period, the level of microcirculation in rats treated with ZnO NPs–triterpenoid oleogels almost returned to the value characteristic of intact animals, which creates conditions for skin regeneration.

It can be assumed that the increase in the perfusion index during treatment reflects a decrease in the depth of the wound, given the fact that the depth of the initial wound was 3–5 mm. The relationship between an increase in blood flow, and, accordingly, the perfusion index, with wound depth decrease, is reliably confirmed in several studies [32,33].

The perfusion index makes it possible to characterize the depth of a burn wound in vivo. At the same time, it is not always possible to obtain a reliable value of the wound depth from histological studies carried out posthumously, especially if wound depth is uneven.

#### 2.2.3. The Enzyme Activity

The enzyme activity under the action of ZnO NPs oleogels at the dose of 25 mg∙cm^−2^ per day was studied after 10 days of treatment on rats. Data in Table 6 indicate the decrease in malonic dialdehyde (MDA) level as well in plasma as in erythrocytes under the action of ZnO NPs oleogels.

This fact is very unexpected and interesting because ZnO NPs usually cause the generation of reactive oxygen species [34,35,36]. It can be assumed that in a lipophilic medium in the presence of lipophilic triterpenoids, the mechanism of action of nanoparticles on cells will be different from the pro-oxidant effect and decrease lipid peroxidation in the organism.

Moreover, the treatment by ZnO NP oleogels activates the antioxidant metabolic processes, mainly antioxidant enzyme activity (superoxide dismutase, catalase, glutathione reductase and glucose-6-phosphate dehydrogenase), in rats (Table 7).

The cuprum-zinc-superoxide dismutase (Cu-Zn-SOD) shows the most significant effect on oxidative stress because it removes the superoxide anion. This enzyme has high stability [37]. CuZn-SOD catalyzes the dismutation of constantly emerging at anaerobic metabolism superoxide into oxygen and hydrogen peroxide. In the human body, enzymes included cuprum and zinc act synergically and with the third element, selenium, that comprises a uniform antioxidant defense. That is why the research of antioxidant defense is most appropriate with regard to the activity of enzymes, such as glutathione peroxidase (containing selenium) and glutathione reductase, as well as considering the concentration of glutathione.

The role of zinc, which does not change the valency against oxidative stress, is more complicated than that of copper interacting, as in the Fenton reaction. In this context, zinc may be considered as an “indirect” antioxidant—as a modifier of biological redox-reactions. The antioxidant effect of zinc is mainly due to its ability to initiate the stress reaction in terms of (1) stimulation of MTF-1-dependent transcription and (2) activation of stress-sensitive signal cascades MAPK and PI3K/Akt. Besides, the antioxidant effect of zinc associates with stabilization of protein thiols (enzymes, “zinc fingers”, metalloproteins) [37].

One of the potential mechanisms of antioxidant action of zinc is next. Zinc can be an antagonism of oxidation-reduction transition metals such as iron or cuprum and also prevents oxidation of sulfhydryl groups in proteins. Thiol groups are stabilized by zinc, and that protects the enzyme or other protein from inactivation caused by oxidative stress. Zinc antioxidant effect, in case of metalloproteins, is due to the regulation of their metabolism. In turn, zinc deficiency leads to declining of sulfhydryl groups defense and also increases the production of reactive oxygen species (ROS). Zinc in high concentration can be pro-oxidants, causing drop the level of CuZn-SOD and other main enzymes in erythrocytes.

LDH activity was noted to be increased, especially under the action of ZnO NP compositions that also led to decrease in lactic acid resulting in the decrease in hypoxia in burn trauma (the maximum effect at ZnO NPs-BA oleogel usage) (Table 7).

## 3. Materials and Methods 

### 3.1. Materials

Betulin was purchased from Sigma-Aldrich (CAS 473-98-3). FTIR, ν, cm^−1^: 3470 st (OH), 1640 st (C=C); ^1^H-NMR δ, ppm: 4.67 m (1H, =CH_2_), 4.57 m (1H, =CH_2_), 3.78 br. s (1H, 28-CH_2_OH), 3.31 m (1H, 28-CH_2_OH), 3.17 m (1H, 3-CHOH), 2.36 m (1H, 19-CH), 1.66 s (3H, CH_3_), 1.23 s (3H, CH_3_), 0.96 s (3H, CH_3_), 0.94 s (3H, CH_3_), 0.80 s (3H, CH_3_), 0.74 s (3H, CH_3_). ^13^C-NMR, δ, ppm: 76.71 (C-3), 109.46 (C-29), 150.24 (C-20), 57.87 (C-28). Spectral data are presented in Figure S2.

Betulin-3,28-diphosphate (BDP, 3β,28-diphosphate-lup-20(29)-ene) was synthesized according to the procedure [38]. FTIR (KBr): ν_max_ 3421, 2331, 2342, 1641-1700, 1240, 1031, 973, 501 cm^−1^; ^1^H-NMR (DMSO-*d*_6_, 400 MHz) δ 0.68–1.99 (42H, m, 6CH_3_, (CH_2_)_10_, (CH)_4_), 2.35–2.42 (1H, m, H-19), 2.97 (0.25H, wide t, α-H-3, *J* = 7.7 Hz), 3.69 (0.75H, ddd, β-H-3 m, *J* = 4.6, 7.8, 11.2 Hz), 3.96 (1H, dd, H-28, *J* = 9.7, 4.5 Hz) and 3.52 (H, dd, H-28′, *J* = 9.5, 4.5 Hz), 4.55, 4.69 (2H, two s, H-29), 5.69 (protons in the phosphate groups O-P(O)(OH)_2_, wide blurred s); ^13^C-NMR (DMSO-*d*_6_, 101 MHz) *δ*, ppm: 149.93 (C, C-20), 109.92 (CH_2_, C-29), 82.96 (CH, =CHOH), 63.22 (CH_2_, CH_2_OH), 54.90 (CH, C-5), 49.71 (CH, C-9), 48.11 (CH, C-19), 47.26 (CH, C-18), 46.75 (C, C-17), 42.30 (C, C-14), 40.48 (C, C-8), 38.57 (C, C-4), 38.35 (CH_2_, C-1), 37.99 (C, C-10), 37.07 (CH, C-13), 36.57 (CH_2_, C-7), 33.79 (CH_2_, C-22), 29.11 (CH_2_, C-21), 28.96 (CH_2_, C-16), 28.17 (CH_3_, C-23), 27.90 (CH_2_, C-2), 26.57 (CH_2_, C-15), 24.83 (CH_2_, C-12), 20.43 (CH_2_, C-11), 18.84 (CH_3_, C-30), 18.01 (CH_2_, C-6), 16.14 (CH_3_, C-26), 15.91 (CH_3_, C-24), 15.70 (CH_3_, C-25), 14.56 (CH_3_, C-27); ^31^P-NMR (DMSO-*d*_6_, 202.46 MHz) δ −0.4 (d, *J* = 8.2 Hz, phosphoric acid residue at C-3β), 0.48 ppm (t, *J* = 4.6 Hz, phosphoric acid residue at C-28). Spectral data are presented in Figures S2–S5.

Betulin-3,28-diacetate (BDA, 3β, 28-diacetate-lup-20(29)-ene) was prepared according to the procedure [39]. FTIR (KBr): ν_max_ 3068,75 (C=C); 2947,23; 2870,08 (C-H); 1737,86 (C=O); 1456,26; 1388,75; 1365,60 (C-C); 1244,09; 1031,92 (C-O-C), 979,84 (C-O), 889,18 (C=C); ^1^H-NMR (CDCl_3_) δ, ppm: 4,72 (1H, m, =CH_2_); 4,55 (1H, m, =CH_2_); 4,47 (1H, m, H-3); 4,26 (1H, d, J10.7 Hz, H-28); 3,86 (1H, d, J10.7 Hz, H-28); 2,50 (1H, m, H-19); 2,08 (3H, s, CH_3_CO); 2,03 (3H, s, CH_3_CO); 1,65; 1,02; 0,94; 0,82; 0,80 (3H, s, CH_3_); ^13^C-NMR (CDCl_3_) δ, ppm: 14,95 (C-27), 15,79 (C-24), 15,92 (C-25), 16,27 (C-26), 17,94 (C-6), 18,89 (C-29), 20,58 (C-11), 20,77 (CH_3_AC), 21,04(CH_3_AC), 23,46 (C-2), 24,92 (C-12), 26,83 (C-15), 27,71 (C-23), 29,35 (C-16), 29,52 (C-21), 33,91 (C-7), 34,32(C-22), 36,82 (C-10), 37,32 (C-13), 37,55 (C-4), 38,16(C-1), 40,66 (C-8), 42,45 (C-14), 46,08 (C-17), 47,47(C-19), 48,54 (C-18), 50,05 (C-9), 55,15 (C-5), 62,50 (C-28), 80,61 (C-3), 109,69 (C-30), 149,80 (C-20), 170,63 (COAC), 171,23 (COAC). Spectral data are presented in Figures S2 and S6.

Betulonic acid (BA) was obtained by methods [40]. FTIR, ν, cm^−1^: 1705 st (C=O), 1641 st (C=C); 883 st (=CH_2_); ^1^H-NMR δ, ppm: 4.68 s (1H, 29-H), 4.55 s (1H, 29-H), 2.23 m (1H, 19-H), 1.65 s (3H, 30-CH_3_), 1.02–1.95 (3H, complex, CH_2_, CH), 1.02 s (3H, 26-CH_3_), 1.00 s (3H, 25-CH_3_), 0.98 s (3H, 27-CH_3_), 0.86 s (3H, 23-CH_3_), 0.85 s (3H, 24-CH_3_). ^13^C-NMR δ, ppm: 216.52 (C-3), 109.67 (C-29), 150.33 (C-20), 177.26 (C-28). Spectral data are presented in Figures S2 and S7.

### 3.2. Obtaining of Zinc Oxide Nanoparticles [18,19]

We used freshly prepared 2% solution of sodium hydroxide (or lithium hydroxide) in 96% ethanol (or methanol), and 1.5% solution of zinc acetate dehydrate in 96% ethanol (or methanol) at 70 °C. The solution of sodium or lithium hydroxide (10 mL) was added drop by drop to 30 mL solution of zinc acetate dehydrate in an ice bath and was mixed up for 5–10 min. White flakes generated were precipitated by heptane (60 mL). After the liquid and solid phase separation, the precipitate was washed on a paper filter successively by ethanol and heptane to remove an excess of acetate and sodium ions. The precipitate was dried at 105 ± 5 °C for 5 h.

### 3.3. FTIR Analysis

FTIR spectra in 400–4000 cm^−1^ range were measured by an IR Prestige-21 FTIR spectrometer (Shimadzu, Kyoto, Japan) equipped with a KBr beam splitter. A pellet from a well-dried KBr was prepared according to standard cold pressing. The resolution was 0.5 cm^−1^. The number of scans was 45.

### 3.4. UV Analysis

UV spectra were recorded by UV-1800 (Shimadzu, Kyoto, Japan).

### 3.5. RP-HPLC Analysis

Reversed phase high-performance liquid chromatography analysis was carried out on LC-20Avp (Shimadzu, Kyoto, Japan) with UV-detection, the column is Discovery C18 (25 cm × 4.6 mm, 5 μm, Supelco).

### 3.6. Powder X-ray Diffraction Analysis

Powder X-ray diffraction patterns were obtained using Shimadzu X-ray diffractometer XRD-6000 at 295(2) K with Cu Kα radiation (λ = 1.5418 Å), in the Bragg–Brentano reflection geometry. The samples were collected in the 2θ range between 5 and 50° with steps of 0.026° and 100 s of step size, using a scan speed (°/s) of 0.067335. On the X-ray diffraction patterns of amorphous samples, there are diffraction peaks at 37.5° and 44.0° referring to the cuvette material.

### 3.7. Photoluminescence Analysis

Fluorescent spectra were obtained using spectrofluorimeter RF-600 (Shimadzu, Kyoto, Japan) at the length 320 nm in the field 350–800 nm in a 10 mm thick cuvette.

### 3.8. Pharmaceutical Composition

Betulin was colloidally ground with 20 mL of sunflower oil in the presence of α-tocopherol and ascorbyl palmitate. Zinc oxide nanoparticles were treated with an ethanol solution of triterpenoids for 1 h. After centrifugation and drying (100 ± 5 °C) for several hours, zinc oxide nanoparticles with sorbed triterpenoids were also thoroughly homogenized with 50 mL of sunflower oil under the action of ultrasound (44 kHz) and slowly was introduced into the betulin oil mixture. Sunflower oil was added to the composition up to 100 g using ultrasound action [41].

Assay of all triterpenoids before and after sorption from ethanol solution was performed by reversed-phase HPLC analysis: 210 nm, 40 °C, mobile phase A30%-B70% *v/v* (A—acetonitrile (grade 0), B—buffer solution of KH_2_PO_4_, pH = 6.36), flow 1.0 mL∙min^−1^ (Figure S1).

Assay of ZnO NPs was performed by atomic absorption spectrophotometry or titration by disodium dihydrogen ethylenediamine tetraacetate (indicator: xylenol orange triturate R and hexamethylenetetramine R) after the treatment by acetic acid in accordance with European Pharmacopoeia.

### 3.9. Biological Activity

Male Wistar rats (200–250 g) were involved in the study. The animals were purchased from the Animal Breeding Facilities “Stolbovaya” (Chekhov, Moscow region, Russia). All procedures for maintenance and sacrifice (care and use) of animals were carried out according to the criteria outlined by European Convention ET/S 129, 1986 and directives 86/609 ESC. The animals were handled humanely, kept in plastic suspended cages, and placed in a well-ventilated and hygienic rat house under suitable conditions of room temperature (27 ± 2 °C) and humidity. They were given food and water ad libitum and subjected to a natural photoperiod of 12 h light and 12 h dark cycle. The animals were allowed two weeks of acclimatization before the commencement of all animal model experiments in the study.

All blood taking and withdrawal of animals out of experiment were performed under anesthesia, all efforts being made to minimize suffering.

The study as presented was approved by the Local Ethics Committee of Privolzhsky Research Medical University, Russian Federation (Protocol No. 2 from 20 February 2016).

#### 3.9.1. Modeling of Thermal Burns in Animals

The surface of the animal’s back was burned using electromagnetic radiation from an infrared soldering station YaXunXY865D following the requirements of Good Laboratory Practice for experimental modeling of thermal burns in laboratory animals. We used the regime that causes thermal burns of the deep second degree. The distance of the infrared heater from the animal’s skin was 15 mm, the temperature on the skin in the heating zone was 60 °C, the heating duration was 23 s, the power was 100 W. Under these conditions, infrared radiation penetrates to a depth of 3–5 mm [30,31]. Standard thermal burns had an area of 14.0 ± 0.5 cm^2^.

The body surface area of each animal was evaluated by the weight of the animal using the Mee–Rubner formula [42]. The minimum weight of the rats was 240 g, the maximum was 330 g, the average weight of the rats was 285.0 ± 5.0 g. The minimum body surface area of the experimental animals was 436.0 cm^2^, the maximum was 555.0 cm^2^, and the average was 494.0 cm^2^.

#### 3.9.2. Wound Area Measurement

The wound area was measured using a planimetry system. A 2-layered transparent film was placed on the wound, and the outline traced onto the film using a permanent marker. The layer of the film in contact with the wound was discarded. The top layer containing the tracing was retraced onto the graph paper; the wound area was calculated.

After the measurement, the wound contraction was expressed as the percentage change in the original wound area using the following formula (3) [43]:

(3) $$\mathrm{Wound}\;\mathrm{contraction}=\frac{\left(\mathrm{Specific}\;\mathrm{day}\;\mathrm{wound}\;\mathrm{area}\right)\cdot 100\%}{\left(\mathrm{Original}\;\mathrm{wound}\;\mathrm{area}\right)}$$

#### 3.9.3. Biological Activity In Vitro

Biological analysis in vitro was performed using blood stabilized with sodium citrate. Erythrocytes were washed twice with 0.9% NaCl by centrifugation for 10 min at 1600g. The intensity of lipid peroxidation (LPO) was estimated by the MDA level in plasma and erythrocytes following the methods by Uchiyama and Mihara [44]. Superoxide dismutase activity (EC 1.15.1.1) was measured in erythrocytes using inhibition of adrenaline auto-oxidation [45]. Catalase activity (EC 1.11.1.6) was determined by spectrophotometry based on the decomposition of hydrogen peroxide by the catalase [46]. Glutathione reductase activity (EC 1.8.1.7) was studied by spectrophotometry based on the oxidized glutathione reduction [47]. The activity of glucose-6-phosphate dehydrogenase (EC 1.1.1.49) was determined in hemolysate of erythrocytes by spectrophotometry based on glucose-6-phosphate oxidation to the phosphoglucolactone with the formation of reduced nicotinamide adenine dinucleotide phosphate (NADPH) [48]. The energy metabolism in erythrocytes was studied using the catalytic activity of LDH (LDH, EC 1.1.1.27) in direct (LDH_direct_, substrate—50 mM sodium lactate) and reverse (LDH_reverse_, substrate—23 mM sodium pyruvate) reactions [49]. The specific activity of the enzymes was calculated from the protein concentration analyzed by the modified Lowry method [50].

#### 3.9.4. Morpho-histology Research

Morpho-histology research of excise samples of wound tissues was made on samples fixed in 10% solution of neutral formalin. Then, the test material was washed in running water and dehydrated and serial processing in ethanol solutions with the increase in the concentration: 50%, 60%, 70%, 80%, 90%, 96%, 100% for the 40 min in each solution. Dehydrated samples were soaked initially in xylene for 30 min and then in paraffin within 2 h. At the final stage, the samples were poured with molted paraffin. A sled microtome (Chuvash State University named after I.N. Ulyanova, Russia) was used for getting paraffin blocks slices. The samples were colored by hematoxylin and eosin.

#### 3.9.5. Microcirculation Research

The microcirculation was assessed quantitatively using the LAKK-02 (LASMA, Moscow, Russia). This device transmits continuous wave laser light (30 mW, 890 nm) and white light (20 W, 500–900 nm) to skin tissue near the wound, where it is scattered and collected on the skin surface with fibers of the probe. The movement of erythrocytes causes a Doppler shift, which in turn is detected by the laser light and analyzed by the LAKK-02, that is then computed and displayed as the blood flow velocity. The detected laser signal correlates with the number of moving erythrocytes in tissue, blood flow velocity for calculation microcirculation parameters, using such arbitrary (relative) units as perfusion units (perf. un.). The rate of microcirculation (the microcirculation level), the regulatory activity of its components and the degree of shunt paths participation with an allowance for the frequency range intervals of the blood flow oscillations in the rats’ microvessels were investigated [51,52].

### 3.10. Statistical Analysis

Statistical data processing was performed by the software (Statistica 6.0 (StatSoft Inc., Tulsa, OK, USA)). The normality of a distribution of results was shown using the Shapiro–Wilk test. The significance of differences between groups was assessed using Student’s *t*-test and one-way analysis of variance (ANOVA). The differences were considered statistically significant at *p* < 0.05.

## 4. Conclusions

Oleogels containing zinc oxide nanoparticles with sizes 10–20 nm and triterpenoids (betulin, betulonic acid, betulin diacetate and betulin diphosphate) in sunflower oil are useful for the treatment of deep II-degree burns. The active pharmaceutical ingredients, ZnO NPs modified by betulin and its derivatives (betulonic acid, or betulin diacetate, or betulin diphosphate), were characterized. Identification and assay of ZnO NPs were developed by PXRD, FTIR, UV-vis, PL and AAS. Identification and assay of initial triterpenoids were controlled by ^1^H, ^13^C NMR, FTIR, UV–vis spectroscopy as described in the literature [38,39,40].

The effect of oleogels on the treatment of deep II-degree burns was studied on rats using histological studies, Doppler flowmetry and evaluation of enzymes activity and MDA level. On the 10th day of treatment by oleogels, the burn wound area was decreased twice compared to the initial burn area (by 45–55%). The bottom of the burn wound was cleared of scab residues and filled with granulations. In contrast, at the same time, the burn wound area increased by 10–15% in the untreated burned group, and the scab remained.

The microcirculation index in the near-wound zone by 20–30% improved compared with the control group without treatment. Evaluation of the enzyme activity and the MDA level after treatment by oleogels during the 10 days showed them returning to normal. The improvement of antioxidant biochemical indexes, as well as wounds’ healing, was mainly determined by zinc ion influence.

Generally, biochemical research of the different types of burns and dermatological diseases confirmed the optimal zinc level as the necessary condition for maintaining optimal oxidative metabolism [5,53].

Thus, the new pharmaceutical composition of zinc oxide nanoparticles and triterpenes for the burn treatment provide a synergistic effect on the animal’s body—improves regeneration of skin tissue, creates the necessary conditions for wound healing in general, and improves the overall antioxidant status of the body.

## Figures and Tables

**Table 1:** Powder XRD patterns data of zinc oxide nanoparticles modified by triterpenoids (Figure 2) ^1^.

Nanoparticles ^2^	Peaks	β‘, 2θ	β, rad	2θ, °	Cosθ	D, nm
ZnO NPs	100	0.468	0.0082	36.22	0.950	17.8
	002	0.545	0.0082	47.54	0.911	16.0
	110	0.625	0.0109	56.62	0.878	14.5
ZnO NPs-B	101	0.595	0.0103	31.68	0.959	17.3
	002	0.476	0.0083	34.34	0.955	17.5
	100	0.595	0.0103	36.42	0.950	14.0
ZnO NPs-BDA	101	0.487	0.0085	31.70	0.959	16.9
	002	0.365	0.0064	34.38	0.956	22.6
	100	0.512	0.0089	36.2	0.950	16.4
ZnO NPs-BA	101	0.730	0.0127	31.64	0.959	11.5
	002	0.487	0.0085	34.38	0.954	17.0
	100	0.714	0.0125	36.16	0.950	11.7
ZnO NPs-BDP	101	0.714	0.0125	31.76	0.959	11.6
	002	0.476	0.0083	34.36	0.954	17.5
	100	0.714	0.0125	36.22	0.950	11.7

^1^ β‘—reflex width at half maximum, 2θ; β—reflex width at half maximum, rad; 2θ—Bragg scattering angle. ^2^ ZnO NPs modified by betulin (ZnO NPs-B), betulonic acid (ZnO NPs-BA), betulin diacetate (ZnO NPs-BDA) and betulin diphosphate (ZnO NPs-BDP).

**Table 2:** FTIR spectra data of ZnO NPs modified by triterpenoids.

Substance	FTIR Spectrum, ν, cm^−1^			
	ZnO	C-O st	C=O st	PO-H comb; PO-H st P=O st; -O-PO(OH)_2_
ZnO	451	-	-	-
B	-	1080, 1028	-	-
ZnO NPs-B	440–480	1032	-	-
BDA	-	1032, 980	1738	-
ZnO NPs-BDA	471	1080, 1033, 980	1739	-
BA	-	1221	1705	-
ZnO NPs-BA	490–450	1385	1706	-
BDP	-	1200–900 (int. comb. bands ^1^ of C-O and P-O)	-	1641 (PO-H comb.); 1193 (P=O st); 983, 973 (PO-H st); 501 (-O-PO(OH)_2_)
ZnO NPs-BDP	500–450	1200–950 (int. comb. bands of C-O and P-O)	-	1640 (PO-H comb.)—more int.

^1^ int. comb. bands: intense combination bands

**Table 3:** Composition of oleogels.

Oleogel (Group, n = 5)	Betulin, m, g	ZnO NPs Triterpenoid		Stabilizer	Sunflower Oil
		Type	m, g		
ZnO NPs	10.0 g in all compositions	ZnO NPs	5.0	Ascorbic acid + α-tokoferol acetate 0.001–0.010 g	up to 100.0 g
ZnO NPs-BDA		ZnO NPs-BDA	5.3		
ZnO NPs-BA		ZnO NPs-BA	5.4		
ZnO NPs-BDP		ZnO NPs-BDP	5.5		

**Table 4:** Burn wound area on the 10th day.

Group	Burn Wound Area, cm^2^ (% of 1st day)
Intact	-
Burned	15.75 ± 0.35 (112.5 ± 2.5%)
ZnO NPs	7.7 ± 0.74 (55 ± 5.3%)
ZnO NPs-BDA	6.6 ± 0.19 (47 ± 1.4%)
ZnO NPs-BA	6.3 ± 0.26 (45 ± 1.9%)
ZnO NPs-BDP	7.3 ± 0.11 (52 ± 0.8%)

**Table 5:** The influence of topical treatment of burn by ZnO NPs–triterpenoid oleogels on microcirculation index (MI) on rats (*p* < 0.001).

Group	Day	MI ^1^		% of Intact
		Mean ± SD, perf. un.	RSD %	
Intact	0	13.54 ± 0.96	7.09	100
Burned ^2^	0	6.18 ± 0.02	0.32	45.64
	10	8.25 ± 0.05	0.61	60.93
ZnO NPs	10	12.66 ± 0.98	7.78	93.50
ZnO NPs-BDA	10	13.07 ± 0.50	3.85	96.53
ZnO NPs-BA	10	12.42 ± 0.87	7.04	91.73
ZnO NPs-BDP	10	10.74 ± 0.47	4.34	79.32

^1^ The microcirculation index (estimated by laser Doppler flowmetry on the 10th day after burn; perf. un.: in perfusion units. ^2^ Burn wound depth was equal to 3–5 mm [30,31].

**Table 6:** MDA level in plasma and erythrocytes under the action of ZnO NPs-betulin derivatives oleogel at the dose of 25 mg∙cm^−2^ per day after 10 days treatment on rats ^1,2^.

	Value is Taken as 100% (Control Group)	MDA Level, % of Control			
		ZnO NPs	ZnO NPs-BDA	ZnO NPs-BA	ZnO NPs-BDP
Plasma	0.90 µmol/L (intact)	50.66 ± 1.46	69.03 ± 3.01	85.88 ± 6.90	87.86 ± 5.70
	1.08 µmol/L (burned)	42.33 ± 0.58	57.67 ± l.96	71.75 ± 8.42	73.41 ± 6.64
Erythrocytes	8.94 µmol/L (intact)	60.14 ± 4.74	66.33 ± 5.53	59.35 ± 4.09	57.44 ± 5.06
	11.25 µmol/L (burned)	47.80 ± 3.47	52.72 ± 3.71	47.17 ± 3.56	45.65 ± 3.67

^1^ Number of experiment replications was equal to 3. ^2^ Intact control group: *p* < 0.0001; burnt control group: *p* < 0.001.

**Table 7:** The enzyme activity under the action of ZnO NPs-betulin derivatives oleogel at the dose of 25 mg∙cm^−2^ per day after 10 days treatment on rats (*p* < 0.001).

Enzyme		Biochemical Indexes ^1^			
	Value is Taken as 100% (Control group)	% of Control			
		ZnO NPs	ZnO NPs-BDA	ZnO NPs-BA	ZnO NPs-BDP
SOD	900.76 Ru/mg protein (intact)	71.40 ± 2.12	81.29 ± 3.36	84.57 ± 9.66	77.47 ± 5.40
	597.82 Ru/mg protein (burned)	107.59 ± 5.35	122.48 ± 0.64	127.43 ± 12.87	116.72 ± 10.45
Catalase	30.51 Ru/mg protein (intact)	57.85 ± 2.67	62.81 ± 4.29	79.65 ± 4.37	82.61 ± 5.03
	17.80 Ru/mg protein (burned)	99.18 ± 4.55	107.68 ± 6.56	136.57 ± 10.33	141.63 ± 9.12
GR	90.83 nmol NADH/min/mg protein (intact)	104.81 ± 7.23	78.61 ± 3.09	152.58 ± 9.76	112.75 ± 12.44
	57.44 nmol NADH/min/mg protein (burned)	165.74 ± 6.70	124.30 ± 5.49	241.27 ± 13.45	178.28 ± 15.62
G6PD	40.57 nmol NADPH/min/mg protein (intact)	95.95 ± 8.39	101.90 ± 8.94	107.95 ± 8.56	120.79 ± 8.43
	25.78 nmol NADPH/min/mg protein (burned)	151.01 ± 4.95	160.36 ± 5.23	169.90 ± 4.69	190.11 ± 9.99
LDH_direct_	42.77 nmol NADH/min/mg protein (intact)	75.10 ± 6.74	77.77 ± 4.82	83.61 ± 4.95	73.78 ± 3.78
	25.87 nmol NADH/min/mg protein (burned)	124.18 ± 8.05	128.59 ± 7.33	138.25 ± 8.14	121.99 ± 5.76
LDH_reverse_	174.18 nmol NADH/min/mg protein (intact)	107.02 ± 7.72	87.22 ± 1.98	90.58 ± 3.94	98.06 ± 4.85
	146.49 nmol NADH/min/mg protein (burned)	127.25 ± 9.42	103.71 ± 9.55	107.70 ± 5.62	116.60 ± 9.53

^1^ The number of experiment replications was equal to 3. SOD: superoxide dismutase, GR: glutathione reductase, G6PD: glucose-6-phosphate dehydrogenase, LDH: lactate dehydrogenase, NADH: nicotinamide adenine dinucleotide phosphate.

## Supplementary Materials

The following are available online at https://www.mdpi.com/1424-8247/13/9/207/s1, Figure S1: HPL chromatograms of initial solutions of betulin, betulin diacetate, betulonic acid and betulin diphosphate (insert—HPL chromatograms after sorption on the ZnO NPs surface), Figure S2: FTIR spectra of Betulin, BDA, BA, and BDP, Figure S3: ^1^H-NMR spectrum of BDP. DMSO-d_6_, standard TMS, 400 MHz, Figure S4: ^13^C-NMR spectrum (a) and dept spectrum (b) of BDP (DMSO-d_6_, TMS standard), Figure S5: ^31^P-NMR spectrum of BDP (DMSO-d_6_, standard Ph_3_P), Figure S6: ^13^C-NMR spectrum of betulin diacetate, Figure S7: ^13^C-NMR spectrum of betulonic acid.
