# Supplementary material for: The New Pharmaceutical Compositions of Zinc Oxide Nanoparticles and Triterpenoids for the Burn Treatment
Source: Pharmaceuticals (Basel). 2020 Aug 22;13(9):207. doi: 10.3390/ph13090207 (PMC7558657; doi:10.3390/ph13090207)
Supplement: Supplementary file 1 [file pharmaceuticals-13-00207-s001.zip › pharmaceuticals-887031. Supplementary materials.docx]

**Figure S1:** HPL chromatograms of initial solutions of betulin, betulin diacetate, betulonic acid and betulin diphosphate (insert – HPL chromatograms after sorption on the ZnO NPs surfase)


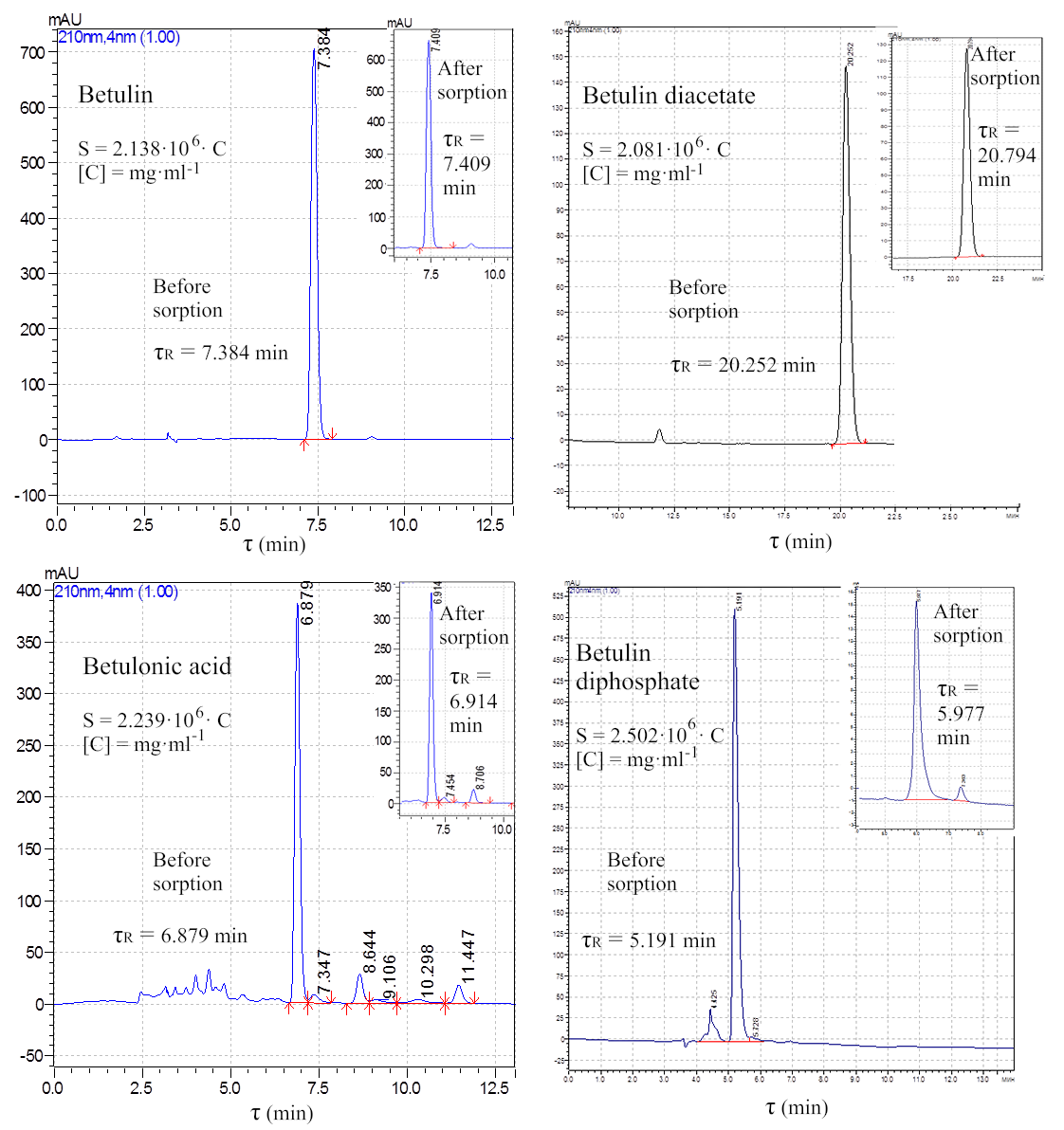


**Figure S2:**  FTIR spectra of Betulin, BDA, BA, and BDP

B

BDA

BDP

BA

**Figure S3:** ^1^H-NMR spectrum of **BDP**. DMSO-d_6_, standard TMS, 400 MHz


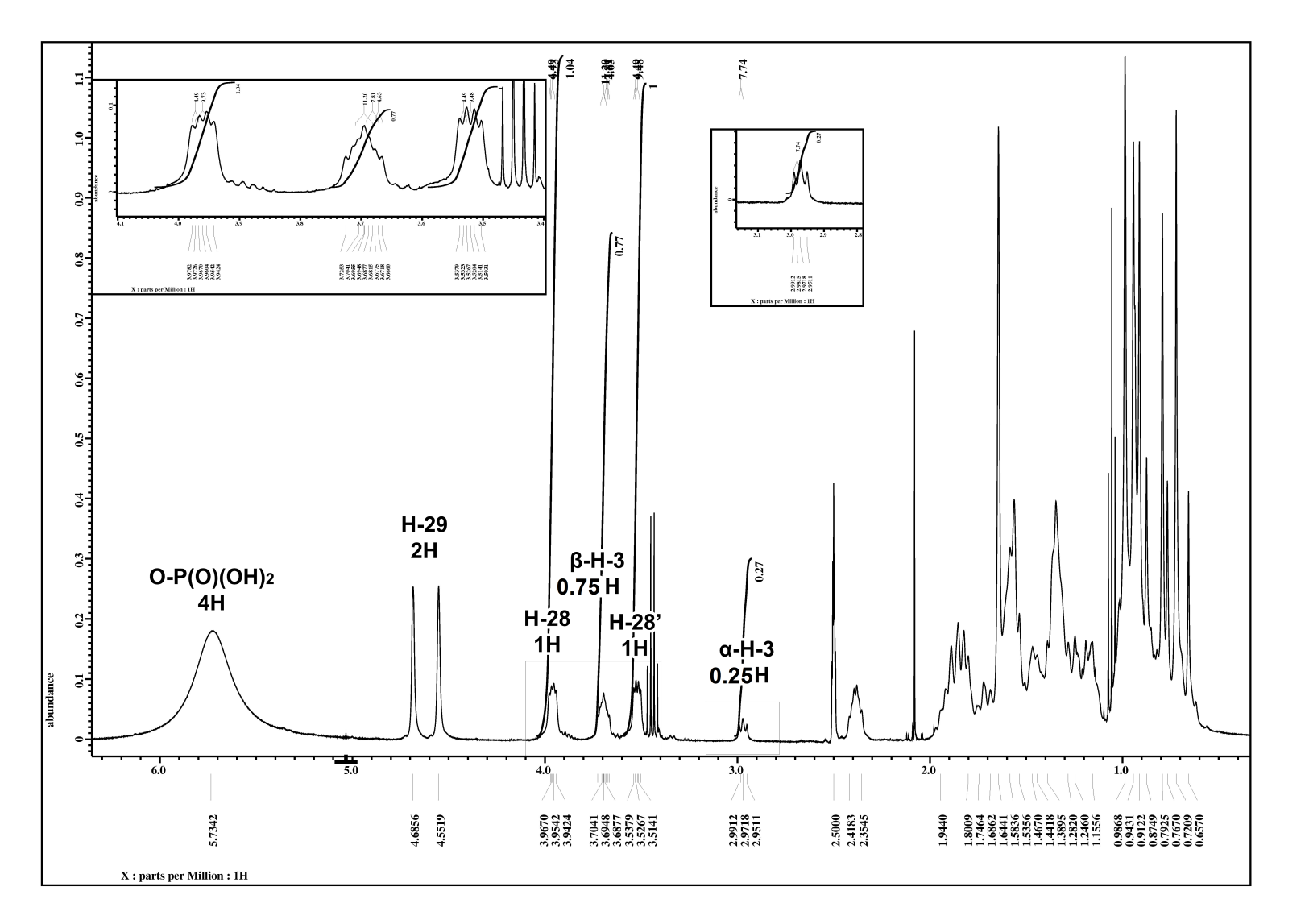


^1^H NMR (DMSO-d_6_, 400 MHz) *δ* 0.68-1.99 (42H, m, 6CH_3_, (CH_2_)_10,_ (CH)_4_), 2.35-2.42 (1H, m, H-19), 2.97 (0.25Н, wide t, α-Н-3, *J* = 7.7 Hz), 3.69 (0.75H, ddd, β-H-3 m *J* = 4.6, 7.8, 11.2 Hz ), 3.96 (1Н, dd, H-28, *J* = 9.7, 4.5 Hz) and 3.52 (Н, dd, H-28’, *J* = 9.5, 4.5 Hz), 4.55, 4.69 (2Н, two s, H-29), 5.69 (protons in the phosphate groups О-Р(O)(ОН)_2_, wide blurred s)

**Figure S4:** ^13^C-NMR spectrum (**a**) and dept spectrum (**b**) of **BDP** (DMSO-d_6_, TMS standard)


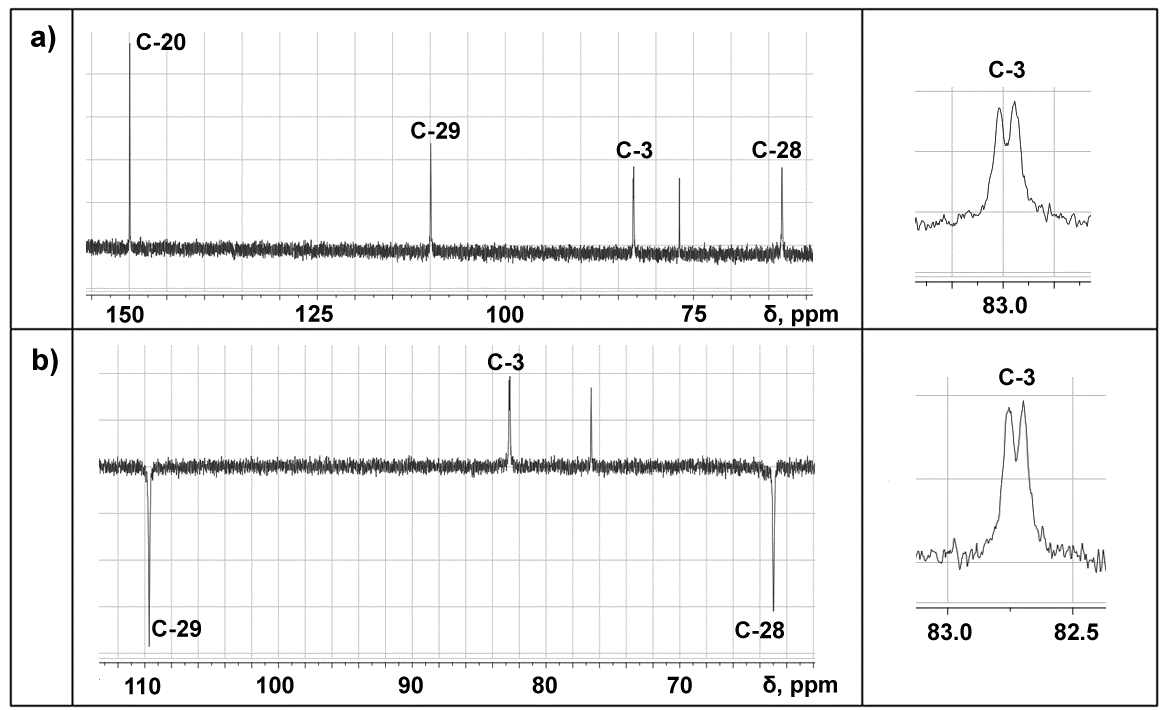


**Figure S5:** ^31^P-NMR spectrum of **BDP** (DMSO-d_6_, standard Ph_3_P)


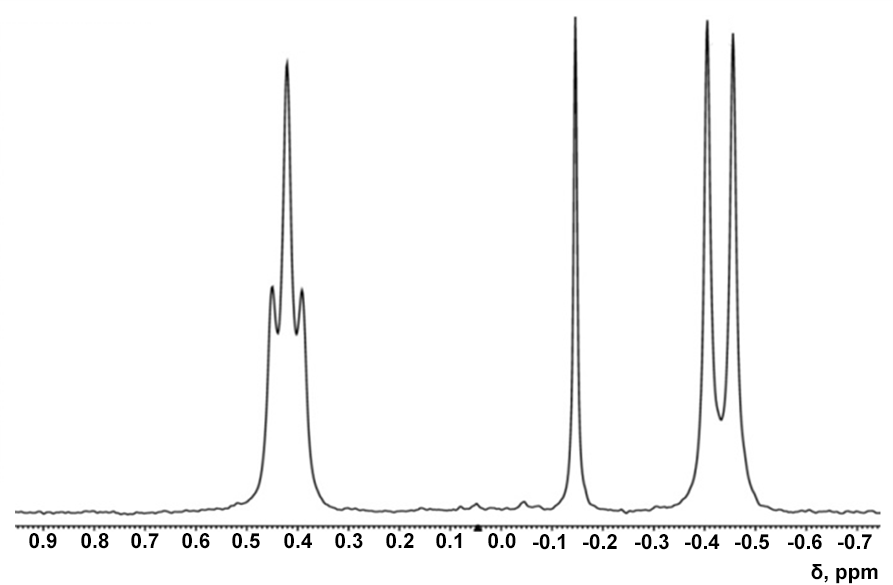


**Figure S6:** ^13^С-NMR spectrum of betulin diacetate


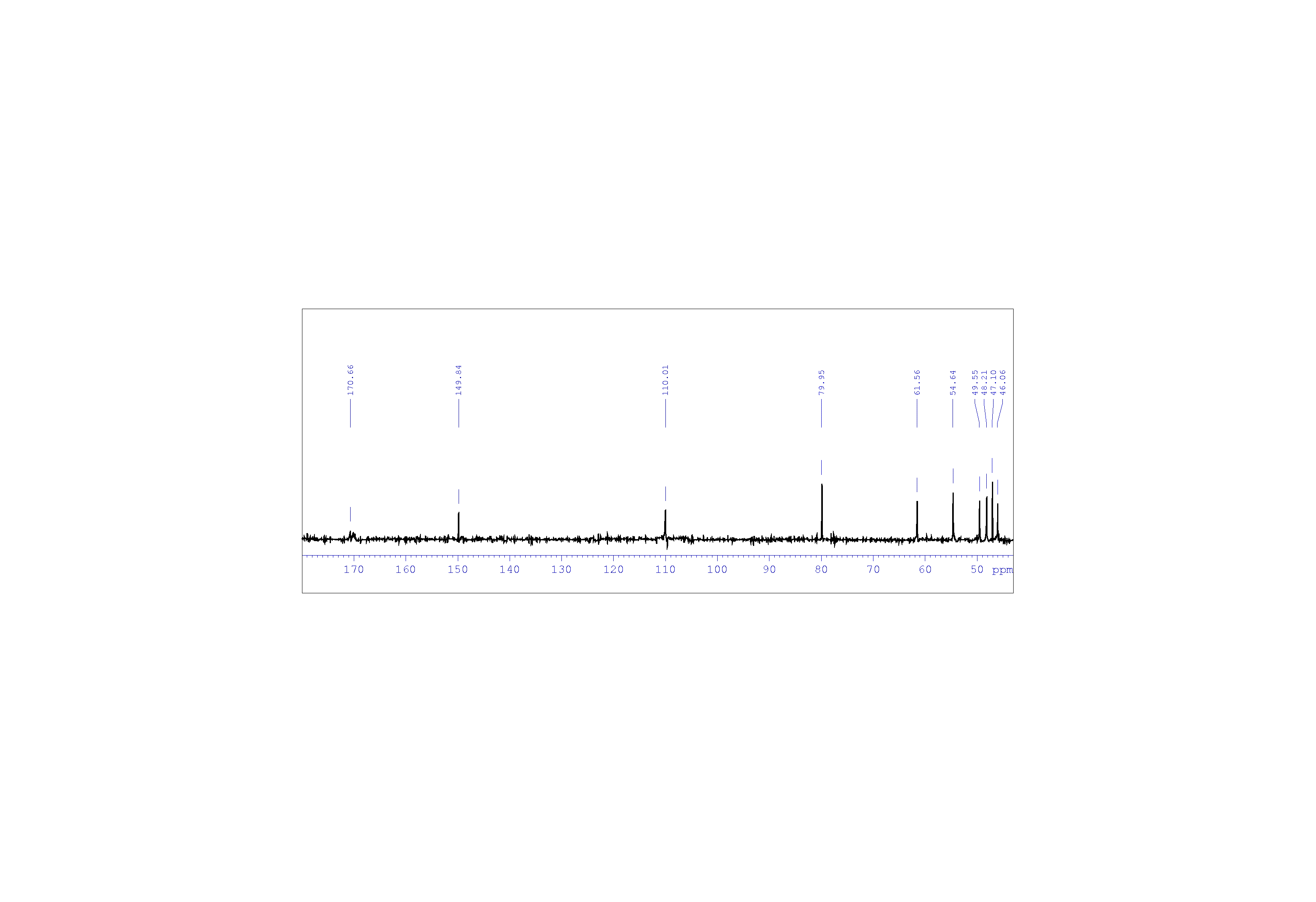


| **Figure S7:** ^13^С-NMR spectrum of betulonic acid   |
| --- |
